# High-Impact Polystyrene Reinforced with Reduced Graphene Oxide as a Filament for Fused Filament Fabrication 3D Printing

**DOI:** 10.3390/ma14227008

**Published:** 2021-11-19

**Authors:** Marta Sieradzka, Janusz Fabia, Dorota Biniaś, Tadeusz Graczyk, Ryszard Fryczkowski

**Affiliations:** Faculty of Materials, Civil and Environmental Engineering, University of Bielsko-Biala, Willowa 2, 43-309 Bielsko-Biala, Poland; jfabia@ath.bielsko.pl (J.F.); dbinias@ath.bielsko.pl (D.B.); tgraczyk@ath.bielsko.pl (T.G.); rfryczkowski@ath.bielsko.pl (R.F.)

**Keywords:** high-impact polystyrene, reduced graphene oxide, composites, filament for 3D printing

## Abstract

Graphene and its derivatives, such as graphene oxide (GO) or reduced graphene oxide (rGO), due to their properties, have been enjoying great interest for over two decades, particularly in the context of additive manufacturing (AM) applications in recent years. High-impact polystyrene (HIPS) is a polymer used in 3D printing technology due to its high dimensional stability, low cost, and ease of processing. However, the ongoing development of AM creates the need to produce modern feedstock materials with better properties and functionality. This can be achieved by introducing reduced graphene oxide into the polymer matrix. In this study, printable composite filaments were prepared and characterized in terms of morphology and thermal and mechanical properties. Among the obtained HIPS/rGO composites, the filament containing 0.5 wt% of reduced graphene oxide had the best mechanical properties. Its tensile strength increased from 19.84 to 22.45 MPa, for pure HIPS and HIPS-0.5, respectively. Furthermore, when using the HIPS-0.5 filament in the printing process, no clogging of the nozzle was observed, which may indicate good dispersion of the rGO in the polymer matrix.

## 1. Introduction

Additive manufacturing (AM), commonly known as 3D printing, has only seen a significant increase in interest for around three decades [1,2]. The International Organization for Standardization (ISO)/American Society for Testing and Materials (ASTM) 52900:2015 defines AM as the “process of joining materials to make parts from 3D model data, usually layer upon layer, as opposed to subtractive manufacturing and formative manufacturing methodologies” [3]. Depending on the printing mechanism, AM processes can be classified into seven categories: binder jetting, directed energy deposition, material extrusion, material jetting, powder bed fusion, sheet lamination, and vat photopolymerization [3]. The most common AM techniques include stereolithography (SLA), selective laser sintering (SLS) or melting (SLM), laminated object manufacturing (LOM), and fused filament fabrication (FFF) [4].

The huge increase in interest in additive manufacturing is the result of the many advantages that this technique has over conventional manufacturing techniques such as subtractive processes, formative processes, and joining processes [5]. The main advantage of 3D printing is the production of elements with complex geometry, which are difficult to obtain using traditional methods. In the case of AM, the complex geometry does not affect the final cost of the part. Additive manufacturing is, therefore, of particular importance in prototyping, small-lot production, and precision manufacturing. Its undeniable advantage is also the wide range of materials suitable for printing. Polymers are considered the most common materials in the 3D printing industry due to their diversity and ease of use in various 3D printing processes. They are used in the form of filaments, resins, reactive monomers, and powders. Nevertheless, metals and alloys, ceramics, and concrete are also used as feedstock materials [6,7,8,9].

Due to its numerous advantages, 3D printing has found application in many areas of our lives. The majority of them certainly include biomedicine (e.g., biomedical implants, engineered tissues and organs, and controlled drug delivery systems), aerospace and the automotive industry, electronics, buildings, and sports [6,10,11,12,13]. In addition, 3D printing has also been playing an important role in the fight against the COVID-19 pandemic. In the last year, several publications have appeared in which the authors indicate a significant increase in interest in additive manufacturing in the context of producing personal protective equipment (PPE) or medical and testing devices [14,15,16].

Fused filament fabrication is one of the most widely used and rapidly developing AM technologies [17]. Its progress can be divided into two categories: process development and material development [18]. Both amorphous and semi-crystalline thermoplastic polymers are used as FFF feedstock materials. The most common polymers used to manufacture the filaments include acrylonitrile-butadiene-styrene (ABS), acrylonitrile-styrene-acrylate (ASA), poly(lactic acid) (PLA), polyamide (PA), polycaprolactone (PCL), and polycarbonate (PC) [19,20,21,22]. Moreover, in recent years, a trend related to the application of recycled polymers in additive manufacturing has been observed [23,24,25,26,27]. N.E. Zander et al. [26] used polyethylene terephthalate (PET) obtained from bottles and containers. The authors argued that the use of waste materials in AM, in the private sector, may lower costs and positively impact sustainable development, providing high-value production from waste plastics.

A polymer gaining more and more attention in additive manufacturing is high-impact polystyrene (HIPS). It is widely used in prototyping as it is characterized by high dimensional stability, low cost, and ease of processing and coloring [28,29]. This polymer is obtained as a result of the free radical polymerization of styrene in the presence of rubbers, usually polybutadiene (PB), to enhance the impact strength and toughness of polystyrene [30,31,32]. However, the continuous development of 3D printing technology results in the need to produce feedstock materials with improved properties [33]. Introducing graphene into the polymer matrix is a solution that may improve the electrical, thermal, and mechanical properties of pure polymers, including HIPS [34,35,36,37,38]. X. Wei et al. [39] were the first to demonstrate the printability of a graphene composite using the fused deposition modeling (FDM) method. The authors described a solvent-based method of obtaining ABS/graphene composites, which can be easily scaled up to industry level duplicated on an industrial scale. They observed increased electrical conductivity of the composite filament. For the composite containing 5.6 wt% of graphene, the electrical conductivity improved by four orders of magnitude compared to the composite containing 0.4 wt% of graphene. ABS/graphene composites for 3D printing applications were also obtained by S. Dul et al. [40], who carried out a solvent-free process consisting of melt compounding and extrusion. Printable polyamide 12/graphene nanocomposite filaments for FDM were prepared by D. Zhu et al. [41]. The filaments were obtained by melt compounding, and then they were used for printing using a commercial FDM 3D printer. The research showed that the thermal conductivity and modulus of elasticity of the 3D-printed PA12/graphene elements increased by 51.4% and 7% compared to compression-molded elements. M.A. Caminero et al. [36] demonstrated that the addition of graphene to PLA did not affect the dimensional accuracy of the printed composite made by the FFF method. The PLA/graphene composites showed the best results in terms of surface texture, especially when considering flat and on-edge orientations of printed elements.

To the best of our knowledge, this is the first article to describe and research printable high-impact polystyrene/reduced graphene oxide composite filaments for fused filament fabrication. The obtained filaments were characterized in terms of morphology and thermal and mechanical properties. The printed parts were made of a material characterized by the best parameters, using a commercial FFF 3D printer (URBICUM SP. Z. O O, Zielonki, Poland). The paper also presents the preliminary test results of the obtained printed parts.

## 2. Materials and Methods

### 2.1. Materials

High-impact polystyrene (HIPS) (POLYSTYRENE IMPACT 3450) (MFR = 7 g/10 min; 200 °C, 5 kg) pellets were supplied by Total Refining & Chemicals (Brussels, Belgium). The following reagents were used to obtain graphene oxide: graphite powder with the grain size < 20 µm (Sigma-Aldrich, Poznan, Poland), and KMnO_4_, 98% H_2_SO_4_, 30% H_2_O_2_ (Chempur S.A., Piekary Slaskie, Poland). The obtained graphene oxide was purified with hydrochloric acid (35–38% HCl, Chempur S.A., Piekary Slaskie, Poland). Reduced graphene oxide was prepared via thermal reduction. The interlayer distance and the average number of layers for rGO were 0.37 nm and 6, respectively. The elemental composition of rGO was 18.4 at.% O and 81.6 at.% C. The method of preparation and detailed characteristics of rGO can be found in the Supplementary Materials (Sections S1 and S2) [42,43].

### 2.2. Sample Preparation

The two-stage process was used to produce the HIPS/rGO filaments. The first step consists in preparing a masterbatch with the addition of 10 wt% rGO. The masterbatch was obtained using a Zamak Mercator EHP-2x16S co-rotating twin-screw extruder (Skawina, Poland). The equipment parameters are 15.8 mm (screw diameter) and the length/diameter ratio of 40. The temperature range from hopper to head ranged from 140 °C to 240 °C. Next, the masterbatch was chopped into pellets and re-extruded to prepare a filament with a uniform diameter of 1.75 mm, which fit the commercialized 3D printer. The composite filaments were obtained with 0.1, 0.5, 1, and 2 wt% content of reduced graphene oxide. 

The tensile test specimens were fabricated using an Urbicum DX 3D printer (URBICUM SP. Z. O O, Zielonki, Poland). The FFF printing parameters are listed in Table 1.

**Figure 1:**
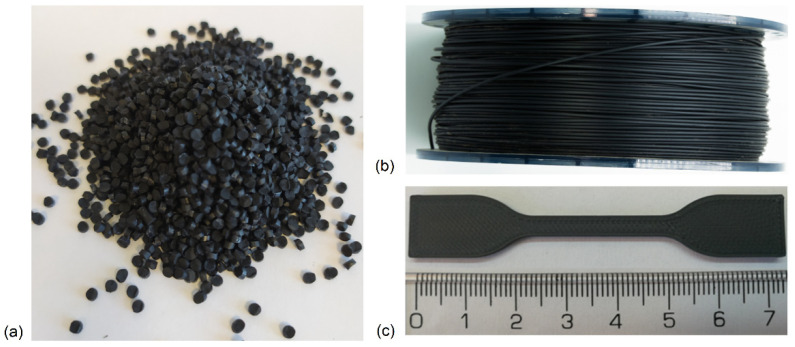
Pellets of a masterbatch containing 10 wt% rGO (**a**); HIPS filament with 0.5 wt% rGO content (**b**); tensile bar HIPS-0.5 (**c**).

Figure 1 shows pictures of the HIPS materials at different production stages.

### 2.3. Methods

A Phenom ProX scanning electron microscope (SEM) (Thermo Fisher Scientific, Watham, MA, USA) was used to observe the morphology of the filaments. Cross-sections samples were prepared by the fracturing of the filaments after cooling in liquid nitrogen. All samples were coated with ~10 nm layer of gold using Leica EM ACE200 sputter coater (Leica Microsystems, Wetzlar, Germany). The energy-dispersive spectrometer (EDS) was applied to analyze the elemental composition of the reduced graphene ox-ide. The measurements were taken at a voltage of 15 kV.

Fourier Transform Infrared Spectroscopy (FTIR) measurements were conducted using a Nicolet 6700 FT-IR spectrometer (Thermo Electron Corp., Madison, WI, USA). The apparatus was equipped with an MTEC model 300 photoacoustic accessory (MTEC Photoacoustics, Inc., Ames, IA, USA). The spectra were registered at the resolution of 8 cm^−1^, the spectral range from 4000 to 400 cm^−1^ with 256 scans and deuterated triglycine sulphate (DTGS) detector. OMNIC software (v. 9.0, Thermo Electron Corp., Madison, WI, USA) was applied to collect data and post-processing.

A differential scanning calorimetry (DSC) was performed using Universal V4.5A TA Instruments (TA Instruments, New Castle, DE, USA). The samples were heated from 0 to 210 °C at a heating rate of 20 °C/min. Measurements were carried out under a nitrogen atmosphere with a purge flow of 40 mL/min. DSC curves were recorded during the first and second (after melting) heating cycles. The glass transition temperatures were determined based on the curves, and the enthalpy relaxation peaks were evaluated.

The thermal properties of the samples were determined by a Thermogravimetric Analyzer Q500 (TA Instruments, New Castle, DE, USA). All measurements were con-ducted at a temperature range of 30–550 °C and a heating rate of 20 °C/min in a nitrogen atmosphere (60 mL/min). The Universal V4.5A software supplied by TA Instruments was used to analysis of DSC and TGA curves.

The melt flow rate (MFR) measurements were performed according to PN-EN ISO 1133-1: 2011 (Procedure A) using a Modular Melt Flow, Model 7023.000 (Instron Ceast, Turin, Italy). The MFR measurements were carried out at 200 °C with an applied load of 5 kg.

The tensile properties of HIPS filaments were tested using an Instron 5544 (with a maximum load force of 5 kN) at a constant temperature of 25 °C and humidity of 50% RH. A gauge length of 50 mm and crosshead speed of 20 mm/min were used to determine the tensile strength, Young’s modulus, and elongation at break. The results are the average of at least 20 samples.

## 3. Results and Discussion

### 3.1. SEM Analysis

**Figure 2:**
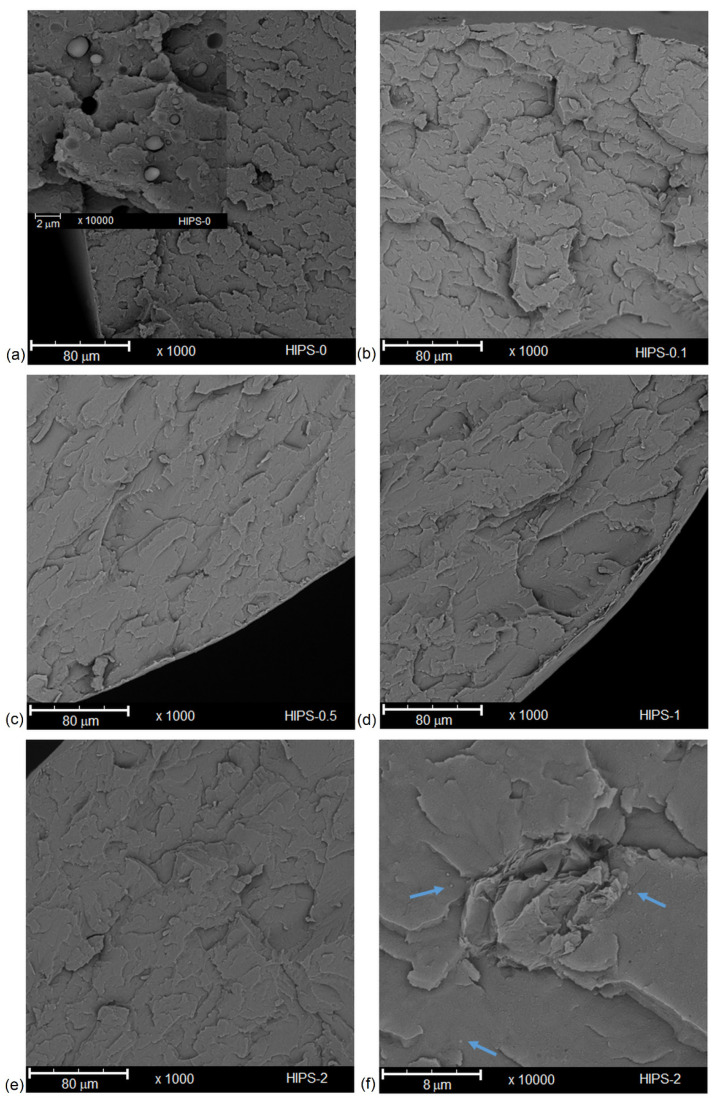
Cross-section of HIPS-0 and HIPS composites: HIPS-0 (**a**); HIPS-0.1 (**b**); HIPS-0.5 (**c**); HIPS-1 (**d**); HIPS-2 (**e**,**f**).

**Figure 3:**
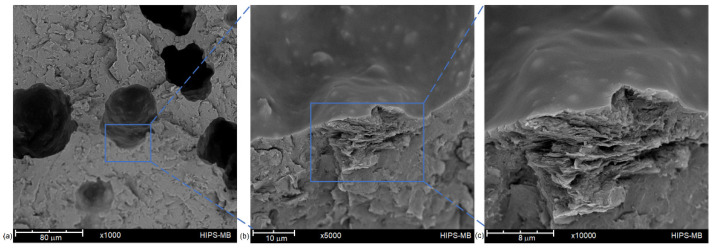
Cross-section of masterbatch filament at different magnifications: ×1000 magnification (**a**); ×5000 magnification (**b**); ×10,000 magnification (**c**).

Figure 2 and Figure 3 show the cross-sectional SEM images of the pure HIPS filament and the HIPS filament reinforced with reduced graphene oxide. The cross-sectional area of all samples at ×1000 magnification is not smooth, and numerous crazes are observed. In the case of the HIPS-0 sample, the polybutadiene rubber particles are clearly seen at high magnification (×10,000, insert in Figure 2a) [44]. These particles are not so numerous for the composite samples and are much smaller (indicated by arrows in Figure 2f). On the cross-section surface of samples containing 0.1 and 0.5 wt% of rGO, no clearly visible agglomerates of this additive are observed. Only at a concentration of 1 and 2 wt% is it possible to notice agglomerates of reduced graphene oxide located both on the edges of the filament and on the surface of the cross-section. This may mean that, at a low concentration of rGO (up to 0.5 wt%), there are no problems with the miscibility of the polymer matrix and the additive [28]. Exceeding this concentration is conducive to the formation of agglomerates. During the extrusion process, it is difficult to attain well-dispersed rGO in the HIPS, especially at higher additive fractions, due to the higher viscosity of composite melts in the presence of rGO and the lack of adequate shear force to break rGO aggregates [45,46]. As presented in further studies, aggregation of the additive affects the properties of the obtained composites.

Figure 3 shows the cross-sectional SEM images of the masterbatch filament. The sample containing 10 wt% of rGO has a completely different morphology from the filament with a lower concentration of reduced graphene oxide. Masterbatch is a porous structure with non-homogeneously scattered pores in the cross-section—numerous fine pores appear around the circumference of the filament. Moreover, observing the cross-sectional surface, many agglomerates of reduced graphene oxide, which influence the mechanical properties of the sample, can be noticed.

### 3.2. Chemical Analysis

**Figure 4:**
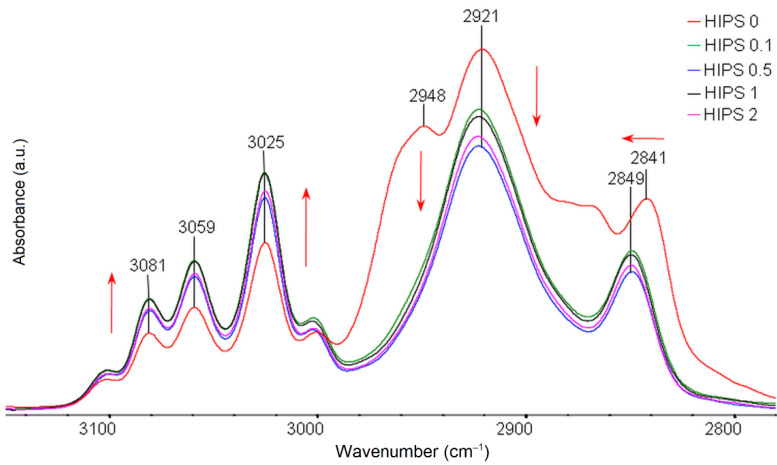
Summary of FTIR spectra for pure HIPS filaments and HIPS filaments with the addition of rGO in a range of 3150–2780 cm^−1^.

**Figure 5:**
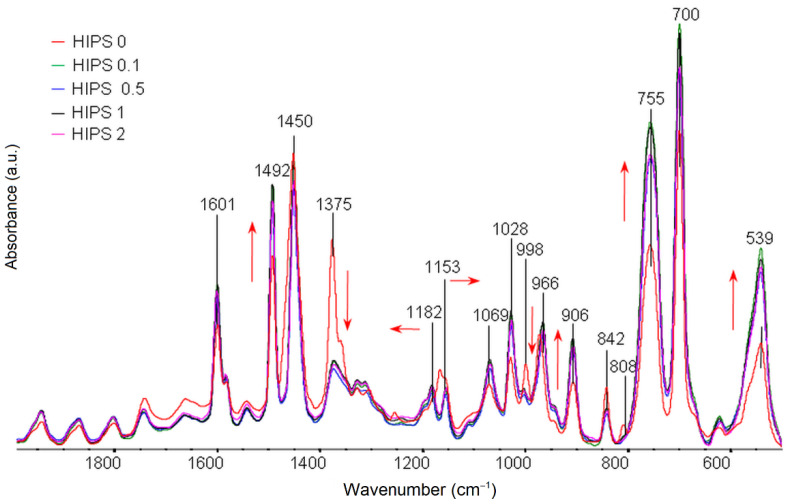
Summary of FTIR spectra for pure HIPS filaments and HIPS filaments with the addition of rGO in a range of 1975–500 cm^−1^.

FTIR test results for pure HIPS filament samples and samples of different rGO content in the range of 3150–2780 cm^−1^ are shown in Figure 4 and for the fingerprint region in Figure 5. Characteristic wavenumber locations of individual oscillators in HIPS are summarized and described in Table 2. Analysis of the obtained FTIR spectra for HIPS samples with different rGO content does not indicate the direct formation of new absorption bands. Nevertheless, it is possible to study the intermolecular interactions between the additive and the polymer matrix by observing changes in characteristic bands (e.g., broadening, intensity) [47]. The spectrum for rGO is characterized by strong absorption of radiation in a wide range of wavenumbers, which makes the bands of rGO oscillators not very intensive. The positions of the maximum bands for the rGO-containing samples remain in the same wavenumber ranges as the band positions in the spectra for pure HIPS. The effect of small amounts of rGO addition (0.1 wt%) on the intensity of the bands is observed. In the spectra (Figure 4), a clear increase in the intensity of the absorption bands is evident at 3081 cm^−1^, 3059 cm^−1^, and 3025 cm^−1^ as a result of radiation absorption by C–H stretching oscillators in the phenyl ring. This indicates a strong interaction between the phenyl substituent ring in HIPS and the rGO rings and probably affects their spatial arrangement to the aliphatic chain. Moreover, changes in the frequency of resonance vibrations of C–H stretching oscillators in the aliphatic chain are observed at 2948–2841 cm^−1^, which may indicate changes in the conformation of the chain within the aromatic substituent under the influence of rGO addition.

In the comparison of FTIR spectra for the fingerprint region (Figure 5), a clear decrease in the intensity of radiation absorption is observed after the addition of rGO at 1375 cm^−1^, which is the result of radiation absorption by the C–H stretching oscillators in the tertiary group. For composites, an increase in the intensity of radiation absorption was also observed at 1182–1069 cm^−1^, originating from deformation vibrations of the C–H oscillator. This may indicate strong spatial interactions between the additive and the matrix, which affect the geometrical arrangement of these oscillators. The single band for the HIPS filament without the addition of rGO visible at 1169 cm^−1^, resulting from the deformation vibrations of the C–H oscillator in the ring, is divided into two bands of different resonant frequencies at 1182 cm^−1^ and 1153 cm^−1^.

### 3.3. Flowability

**Figure 6:**
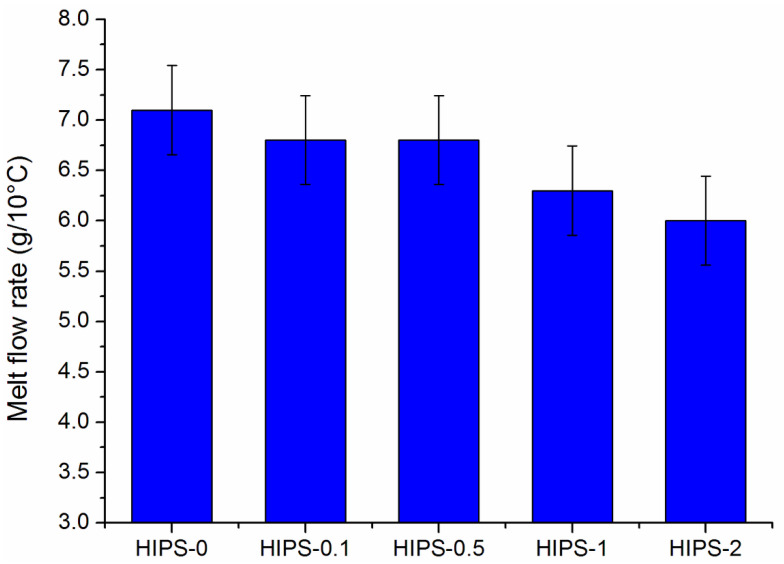
Melt flow rate (MFR) of the pure HIPS filament and the HIPS/rGO composite filaments.

In the case of FFF 3D printing processes, it is crucial to know the extrusion temperatures, as well as the flowability of the printed materials, as this can lead to greater accuracy and precision of the printing process [49]. The melt flow rate (MFR) measurements were used to determine the flowability of the HIPS and HIPS/rGO filaments. The MFR values of the rGO-containing filaments were slightly lower than those of pure HIPS at the temperature applied (Figure 6). Similar results have been reported for other polymers, for which the addition of graphene decreased the MFI of composites together with increasing graphene content [40,41]. This phenomenon is related to the presence of reduced graphene oxide, which limits the mobility and disrupts the flowability of HIPS chains, as further confirmed by thermal analysis.

### 3.4. Thermal Analysis

**Figure 7:**
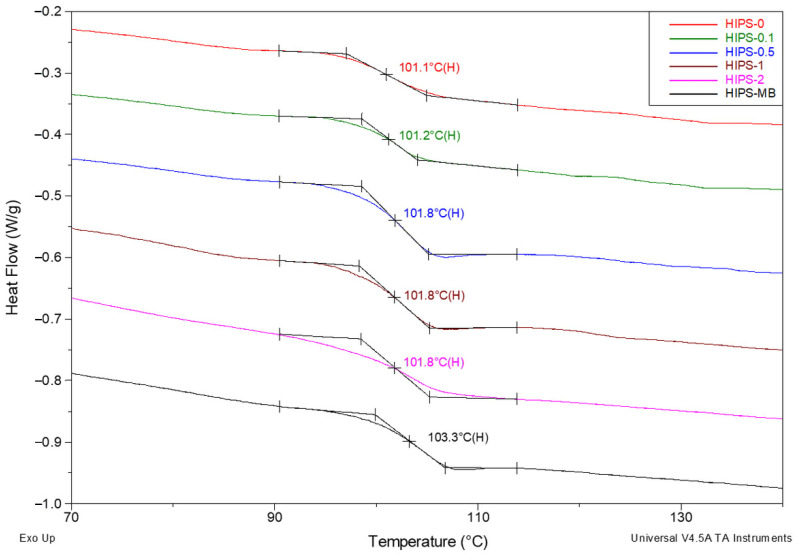
Summary of DSC curves for a series of HIPS filaments registered in the first heating cycle between 70 and 140 °C. Measurements carried out under nitrogen atmosphere, purge flow 40 mL/min. Heating rate 20 °C/min. Analysis of the area of thermal glass transition.

DSC calorimetric tests of HIPS filaments with the addition of reduced graphene oxide were performed in the range of 0.1 to 2 wt%. In order to emphasize the tendency of the observed changes, a masterbatch containing 10 wt% of rGO was also tested. Measurements were carried out on the samples as received, as well as after melting. In both cases, only the glass transition of the HIPS matrix is visible in the DSC curves. The transition is reflected as a characteristic line jump of the calorimetric signal corresponding to a surge in specific heat values in the glass transition temperature range. The glass transition area analysis for HIPS filament samples is shown in Figure 7.

**Figure 8:**
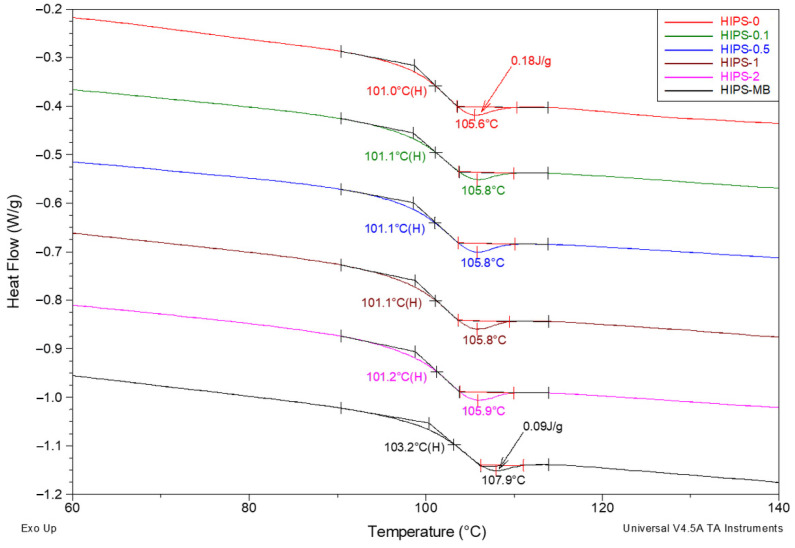
Summary of DSC curves for a series of HIPS samples, created after melting the filaments, registered during the second heating cycle in the range between 40 and 140 °C. Measurements carried out under a nitrogen atmosphere, measurement conditions as above. Analysis of the thermal glass transition area.

As expected, the filament without the addition of rGO (101.1 °C) shows the lowest T_g_ value. However, it should be noted that it is only 0.8 °C lower than the T_g_ for filaments containing 0.5–2 wt% of rGO. The glass transition temperature only for the masterbatch is slightly shifted towards higher temperatures (up to 103.3 °C). The increase in the glass transition temperature shows a decrease in molecular mobility due to filler–matrix interactions [50,51], which confirms the results obtained from MFR measurements. In order to show the impact of the orientation occurring during the formation of the filaments, all the samples were melted. As for the T_g_ values determined based on the DSC curves registered in the second heating cycle, i.e., after melting the filaments (Figure 8), they are only slightly lower than the values determined based on the first scan. The impact of the orientation, therefore, seems to be nearly negligible in the considered case. This can be explained by the relatively large diameter of the filaments (1.75 mm), which, additionally, were not stretched.

The impact of the rGO becomes apparent only when the additional thermal effect accompanying the change in glass transition is considered. It is the endothermic peak of enthalpy relaxation, i.e., so-called apparent melting [52]. This effect is practically invisible in the filament curves (Figure 7), but it appears in all curves for samples created after melting them. Moreover, as the rGO content increases, the minimum temperature of the discussed peak, correlated with the determined T_g_ value, shifts monotonically towards higher temperatures from 105.6 °C for pure HIPS to 107.9 °C for a masterbatch containing 10 wt% of rGO. At the same time, the specific enthalpy of apparent melting decreases in the same order from 0.18 to 0.09 J/g. This clearly proves the influence of the reduced graphene oxide on the HIPS structure. The additive being in the form of rigid graphene micro-sheets hinders the spontaneous arrangement of the structure. This arrangement is destroyed each time the samples are heated to a temperature above T_g_, as a result of which an endothermic peak of apparent melting is formed on the curves.

**Figure 9:**
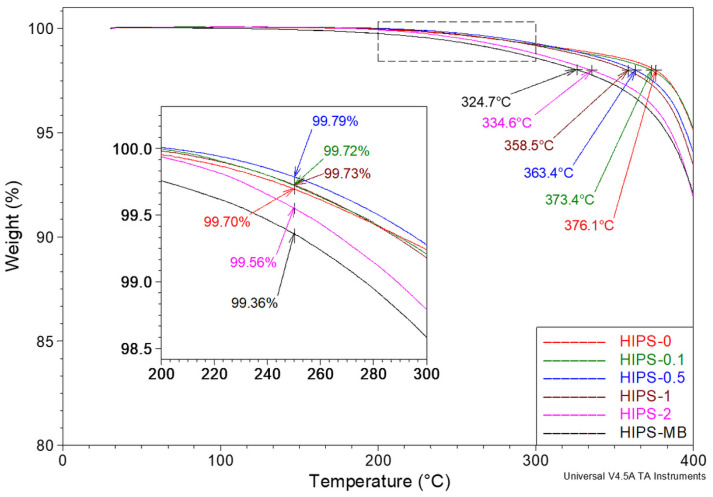
Summary of TG curves for a series of HIPS filaments registered at heating rate of 20 °C/min. Measurements ranging from 30 to 400 °C under nitrogen atmosphere. Purge flow 60 mL/min. The figure shows the 2% mass loss temperature and the analysis of the thermal stability of the samples at 250 °C (insert view).

The discussion of the impact of rGO on the thermal properties of HIPS samples was continued based on the results of thermogravimetric studies. In the first stage of the analysis, the initial shape of the TG curves was studied, and the thermal stability of the samples during heating under a nitrogen atmosphere was determined in two ways (Figure 9). The first method consisted of determining the weight loss of the tested samples at 250 °C, corresponding to the application of the obtained filaments in fused filament fabrication 3D printing (insert view in Figure 9). In the case of the sample without the addition of rGO, the residue at 250 °C is 99.7% of the original weight. Three of the studied samples with relatively low additive content (HIPS-0.1, HIPS-0.5, and HIPS-1) lost slightly less weight than the sample without rGO. Increasing the content of reduced graphene oxide above 1 wt% resulted in a loss of weight that was greater than for the HIPS-0 sample. Nevertheless, the differences are so small (0.4%) that it can be assumed that up to the temperature of 250 °C, the addition of reduced graphene oxide did not affect the thermostability of the polymer. Alternatively, the thermal stability of the test samples was tested by determining the temperature of a 2% weight loss. In this case, a clear trend of changes in thermostability was observed. It was found that this temperature decreased monotonically from 376.1 °C for the pure HIPS sample to 324.7 °C for a masterbatch containing 10 wt% of rGO.

**Figure 10:**
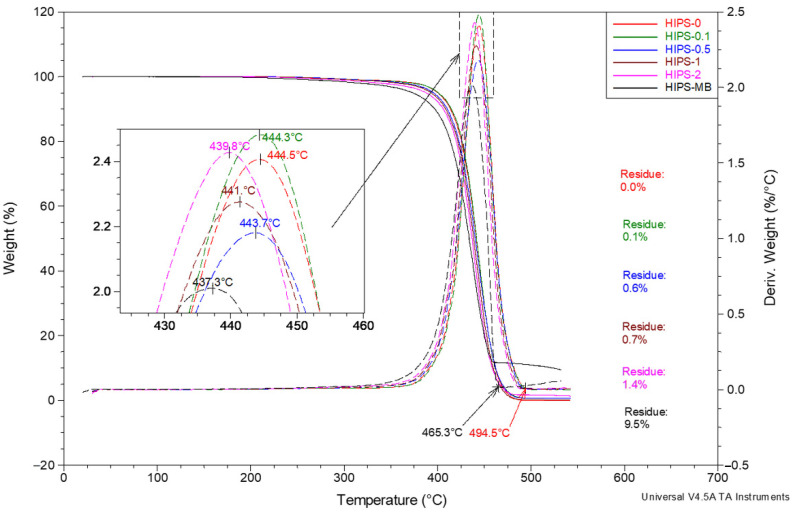
Summary of TG and DTG curves for a series of HIPS filaments registered at heating rate of 20 °C/min. Measurements ranging from 30 to 540 °C under nitrogen atmosphere. Purge flow 60 mL/min. Analysis of the HIPS thermal dissociation area.

The considerations concerning the influence of the reduced graphene oxide on the thermal properties of HIPS based on thermogravimetric studies were also continued at higher temperatures corresponding to the thermal dissociation of the matrix. The analysis of the DTG curves (Figure 10) demonstrated that the temperature of the highest mass loss rate in this transition also decreased monotonically with the increase in rGO content. The corresponding peak maximum on the DTG curve shifts from 444.5 °C for HIPS without additive to 437.3 °C for a masterbatch sample containing 10 wt% of rGO. Moreover, the extrapolated temperature indicated in Figure 10, at which the single-step thermal dissociation of HIPS is completed, is shifted downwards by more than 29 °C for the masterbatch sample compared to the pure HIPS sample. Up to a temperature of approximately 495 °C, the HIPS matrix undergoes complete thermal decomposition. By analyzing the residue after heating all tested samples in nitrogen up to the temperature of 550 °C, high compatibility between this residue and the amount of rGO introduced into the HIPS alloy before forming the filaments was demonstrated.

Based on the thermogravimetric studies, it was demonstrated that the thermal stability of the composite samples was reduced at temperatures above 250 °C. The reduced graphene oxide used in the studies is almost completely stable in the considered temperature range (Figure S1d). A similar phenomenon was observed for a high amount of biocarbon, reducing the thermal stability of its composites [53]. In this case, the reasons for the decreased thermal stability should be sought in the significant increase in thermal conductivity caused by rGO. Because the TGA method used is a dynamic one (the heating rate was 20 °C/min), the samples containing very well heat-conducting graphene were heated up faster in the thermobalance cell, and with relatively high dynamics of measurements, the appropriate temperatures (the beginning, the fastest rate, and the end of thermal decomposition) were registered as shifted in time. Therefore, as a consequence, they were read as lower temperatures. Samples containing reduced graphene oxide, on the other hand, at temperatures up to 250 °C, showed thermal stability similar to that of the pure polymer. This information is essential in the context of using the obtained filaments in fused filament fabrication 3D printing.

### 3.5. Mechanical Properties

HIPS is a polymer with a continuous phase of polystyrene (PS) matrix and dispersed phase of rubber particles with PS occlusions [31]. High-impact polystyrene samples break according to the brittle fracture mechanism with low elongation (they show brittleness). When the HIPS is subjected to stress, many cracks (stress concentration) are generated on the surfaces of the polybutadiene inclusions; PB constitutes a plastic matrix that absorbs the fracture energy. The richer the polybutadiene phase in the polystyrene matrix, the more plastic the material [54]. The tensile strength, Young’s modulus, and elongation at break of the HIPS filaments were determined, and the results are presented in Table 3. The reduced graphene oxide did not significantly affect the strength of the samples. The most remarkable difference in the tensile strength between pure HIPS and the composite HIPS was determined for the sample containing 0.5 wt% of rGO. In this case, the tensile strength increased from 19.84 to 22.45 MPa. It can be noticed that increasing the rGO concentration above 0.5 wt% decreases the tensile strength value. In the case of the Young’s modulus, the same trend of changes was observed. The highest value was determined for the composite containing 0.5% of rGO. The elasticity of samples containing reduced graphene oxide is significantly different from the pure HIPS reference sample. The Young’s modulus for samples containing rGO is similar and ranges from 12.63 to 16.94 GPa, while the Young’s modulus for the pure HIPS sample is 1.21 GPa. M. Amani et al. [55] explained that the increase in tensile modulus can be attributed to the high resistance exerted by the additive against deformation, combined with the stretching resistance of the polymer chains. The elongation of samples in the area of plastic deformation is random, ranging from 0 to 20%; however, for a pure HIPS sample, it is probably related to the brittleness of the material. For the samples with the addition of rGO, the large dispersion of mean values of elongation in this area may also be caused by weak areas related to the agglomerates of the additive. The agglomerates may destroy the adhesion between the components, resulting in a decrease in mechanical properties [56].

### 3.6. Printability

The next stage of research on HIPS filaments reinforced with reduced graphene oxide was checking their suitability for 3D printing. A printed prototype was prepared using a filament that contained 0.5 wt% of rGO (Figure 1c. The HIPS/rGO composite was successfully printed with the same conditions as pure HIPS. During printing, no difficulties, e.g., related to clogging of the nozzle, were observed. SEM studies demonstrated that for the HIPS-0.5 filament, no clearly visible agglomerates were observed, which probably contributed to the fact that the printing proceeded without disturbance related to clogging of the nozzle. While discussing the thermal tests, it was emphasized that the T_g_ of pure HIPS and HIPS/rGO composites differed only slightly. The glass transition temperature also did not change after remelting, i.e., under conditions comparable to the printing process. For this reason, it is possible to use the same printing parameters for pure HIPS and HIPS/rGO composites. The obtained printed parts were examined in terms of their mechanical properties. Preliminary results for tensile strength, elongation, and Young’s modulus can be found in the Supplementary Materials (Figure S2). The studies show that the addition of reduced graphene oxide did not improve the mechanical properties, but it also did not deteriorate them, as the measured strength parameter values are similar. Further work on the HIPS/rGO filament will concern the selection of print parameters to improve the mechanical properties and check other properties of printed parts, i.e., electrical properties.

## 4. Conclusions

In this paper, a method of obtaining filaments from high-impact polystyrene with the addition of reduced graphene oxide for fused filament fabrication (FFF) 3D printing applications was described. HIPS/rGO composite filaments were prepared in a two-step process by melted extrusion using a twin-screw extruder. The filaments were characterized in terms of morphology and thermal and mechanical properties.

Based on the analysis of the SEM images, it was demonstrated that the use of the additive in a concentration above 0.5 wt% causes the formation of rGO agglomerates. Their presence affects the properties of the obtained filaments, contributing, among others, to a decline in tensile strength. The glass transition temperature of the pure polymer and composite filaments differs slightly, which means that it is possible to use the same printing parameters for pure HIPS and HIPS composites. The thermogravimetric studies show that above 250 °C, the obtained composites are characterized by lower thermal stability as compared to pure high-impact polystyrene. However, below this temperature, i.e., within the printing parameters, filaments containing reduced graphene oxide exhibit thermal stability close to that of the pure polymer. Among the obtained HIPS/rGO composites, the filament containing 0.5 wt% of reduced graphene oxide was characterized by the best mechanical properties. For these reasons, the printability of this composite was verified. The HIPS-0.5 filament was successfully printed. The production of a filament containing reduced graphene oxide presents an opportunity for expanding the application of this additive due to the wide interest in additive manufacturing in many areas of our lives.

## Figures and Tables

**Table 1:** Parameters used for the FFF 3D printing of pure HIPS and HIPS-0.5 filaments.

Parameters	Value
Nozzle diameter (mm)	0.5
Nozzle temperature (°C)	250
Bed temperature (°C)	100
Layer height (mm)	0.2
Print infill (%)	100
Print speed (mm/s)	100

**Table 2:** Characteristic wavenumbers of the bands observed in the IR assignments for studied samples [48].

Sample	IR (v cm^−1^) Filament	Assignments
HIPS-0 HIPS-0.1 HIPS-0.5 HIPS-1 HIPS-2	3081	C–H str. ^1^ vibrations of the ring
	3059	C–H str. vibrations of the ring
	3025	C–H str. vibrations of the ring
	2923	C–H str. in CH_2_
	2841; 2849	C–H str. in tertiary groups
	1943	overtone bands in aromatic rings
	1870	overtone bands in aromatic rings
	1803	overtone bands in aromatic rings
	1744	overtone bands in aromatic rings
	1665	overtone bands in aromatic rings
	1601	C–C str. vibrations of the ring, C–H str. in CH_2_ groups acyclic
	1492	C–C str. vibrations of the ring, C–H str. in CH_2_ groups acyclic
	1451	C–C str. vibrations of the ring, C–H str. in CH_2_ groups acyclic
	1375	C–H str. in tertiary groups
	1327	C–H def. ^2^ in rings
	1182	C–H def. in rings
	1155	C–H def. in rings
	1069	C–H def. in rings
	1028	C–C def. in linear alkanes
	966	C–C def. in linear alkanes
	907	C–C def. in linear alkanes
	842	C–H def. out of the plane
	755	C–H def. out of the plane
	700	C–H def. out of the plane
	622	C–H def. out of the plane
	539	C–C in alkane skeletal linear

^1^ str.—stretching, ^2^ def.—deformation.

**Table 3:** Mechanical properties of tested samples.

Samples	Tensile Strength (MPa)	Young’s Modulus (GPa)	Elongation at Break (%)
HIPS-0	19.84 ± 1.25	1.21 ± 0.05	19.74 ± 3.2
HIPS-0.1	19.12 ± 0.57	13.74 ± 0.37	10.04 ± 2.24
HIPS-0.5	22.45 ± 0.88	16.94 ± 0.22	21.9 ± 2.92
HIPS-1	20.48 ± 1.35	12.63 ± 0.41	8.48 ± 2.46
HIPS-2	19.50 ± 0.47	14.18 ± 0.31	20.06 ± 2.12

## Data Availability

Not applicable.

## Supplementary Materials

The following are available online at https://www.mdpi.com/article/10.3390/ma14227008/s1, Figure S1: Characterization of reduced graphene oxide: WAXS pattern (a), FTIR spectra (b), EDS analysis (c), TG curve (d), Figure S2: Picture of HIPS-0 and HIPS-0.5 tensile bars (a), the value of tensile strength (b), Young’s modulus (c), and elongation at break of printed parts (d), Section S1: Preparation of samples, Section S2: Characterization of reduced graphene oxide, Section S3: Mechanical properties of printed parts.

## References

[1] Lopes A.J., Perez M.A., Espalin D., Wicker R.B. (2020). Comparison of ranking models to evaluate desktop 3D printers in a growing market. Addit. Manuf..

[2] Przekop R.E., Kujawa M., Pawlak W., Dobrosielska M., Sztorch B., Wieleba W. (2020). Graphite modified polylactide (PLA) for 3D printed (FDM/FFF) sliding elements. Polymers.

[3] Lee J.Y., An J., Chua C.K. (2017). Fundamentals and applications of 3D printing for novel materials. Appl. Mater. Today.

[4] Guo H., Lv R., Bai S. (2019). Recent advances on 3D printing graphene-based composites. Nano Mater. Sci..

[5] Conner B.P., Manogharan G.P., Martof A.N., Rodomsky L.M., Rodomsky C.M., Jordan D.C., Limperos J.W. (2014). Making sense of 3-D printing: Creating a map of additive manufacturing products and services. Addit. Manuf..

[6] Ngo T.D., Kashani A., Imbalzano G., Nguyen K.T.Q., Hui D. (2018). Additive manufacturing (3D printing): A review of materials, methods, applications and challenges. Compos. Part B Eng..

[7] DebRoy T., Wei H.L., Zuback J.S., Mukherjee T., Elmer J.W., Milewski J.O., Beese A.M., Wilson-Heid A., De A., Zhang W. (2018). Additive manufacturing of metallic components—Process, structure and properties. Prog. Mater. Sci..

[8] Li N., Huang S., Zhang G., Qin R., Liu W., Xiong H., Shi G., Blackburn J. (2019). Progress in additive manufacturing on new materials: A review. J. Mater. Sci. Technol..

[9] Mohan M.K., Rahul A.V., De Schutter G., Van Tittelboom K. (2021). Extrusion-based concrete 3D printing from a material perspective: A state-of-the-art review. Cem. Concr. Compos..

[10] Valino A.D., Dizon J.R.C., Espera A.H., Chen Q., Messman J., Advincula R.C. (2019). Advances in 3D printing of thermoplastic polymer composites and nanocomposites. Prog. Polym. Sci..

[11] Al-Dulimi Z., Wallis M., Tan D.K., Maniruzzaman M., Nokhodchi A. (2021). 3D printing technology as innovative solutions for biomedical applications. Drug Discov. Today.

[12] Beg S., Almalki W.H., Malik A., Farhan M., Aatif M., Rahman Z., Alruwaili N.K., Alrobaian M., Tarique M., Rahman M. (2020). 3D printing for drug delivery and biomedical applications. Drug Discov. Today.

[13] Aslanzadeh S., Saghlatoon H., Honari M.M., Mirzavand R., Montemagno C., Mousavi P. (2018). Investigation on electrical and mechanical properties of 3D printed nylon 6 for RF/microwave electronics applications. Addit. Manuf..

[14] Tino R., Moore R., Antoline S., Ravi P., Wake N., Ionita C.N., Morris J.M., Decker S.J., Sheikh A., Rybicki F.J. (2020). COVID-19 and the role of 3D printing in medicine. 3D Print. Med..

[15] Choong Y.Y.C., Tan H.W., Patel D.C., Choong W.T.N., Chen C.H., Low H.Y., Tan M.J., Patel C.D., Chua C.K. (2020). The global rise of 3D printing during the COVID-19 pandemic. Nat. Rev. Mater..

[16] Aydin A., Demirtas Z., Ok M., Erkus H., Cebi G., Uysal E., Gunduz O., Ustundag C.B. (2021). 3D printing in the battle against COVID-19. Emergent Mater..

[17] Turner B.N., Strong R., Gold S.A. (2014). A review of melt extrusion additive manufacturing processes: I. Process design and modeling. Rapid Prototyp. J..

[18] Shaqour B., Abuabiah M., Abdel-Fattah S., Juaidi A., Abdallah R., Abuzaina W., Qarout M., Verleije B., Cos P. (2021). Gaining a better understanding of the extrusion process in fused filament fabrication 3D printing: A review. Int. J. Adv. Manuf. Technol..

[19] Vaes D., Van Puyvelde P. (2021). Semi-crystalline feedstock for filament-based 3D printing of polymers. Prog. Polym. Sci..

[20] Ligon S.C., Liska R., Stampfl J., Gurr M., Mülhaupt R. (2017). Polymers for 3D Printing and Customized Additive Manufacturing. Chem. Rev..

[21] Pugliese R., Beltrami B., Regondi S., Lunetta C. (2021). Polymeric Biomaterials for 3D Printing in Medicine: An Overview. Ann. 3D Print. Med..

[22] Jafferson J.M., Chatterjee D. (2021). A review on polymeric materials in additive manufacturing. Mater. Today Proc..

[23] Pinho A.C., Amaro A.M., Piedade A.P. (2020). 3D printing goes greener: Study of the properties of post-consumer recycled polymers for the manufacturing of engineering components. Waste Manag..

[24] Zander N.E., Park J.H., Boelter Z.R., Gillan M.A. (2019). Recycled Cellulose Polypropylene Composite Feedstocks for Material Extrusion Additive Manufacturing. ACS Omega.

[25] Zander N.E., Gillan M., Burckhard Z., Gardea F. (2019). Recycled polypropylene blends as novel 3D printing materials. Addit. Manuf..

[26] Zander N.E., Gillan M., Lambeth R.H. (2018). Recycled polyethylene terephthalate as a new FFF feedstock material. Addit. Manuf..

[27] Baechler C., Devuono M., Pearce J.M. (2013). Distributed recycling of waste polymer into RepRap feedstock. Rapid Prototyp. J..

[28] Katančić Z., Travaš-Sejdić J., Hrnjak-Murgić Z. (2011). Study of flammability and thermal properties of high-impact polystyrene nanocomposites. Polym. Degrad. Stab..

[29] Kumar R., Singh R., Farina I. (2018). On the 3D printing of recycled ABS, PLA and HIPS thermoplastics for structural applications. PSU Res. Rev..

[30] Rovere J., Correa C.A., Grassi V.G., Pizzol M.F.D. (2008). Role of the rubber particle and polybutadiene cis content on the toughness of high impact polystyrene. J. Mater. Sci..

[31] Wang F., Chang L., Hu Y., Wu G., Liu H. (2019). Synthesis and properties of in-situ bulk high impact polystyrene toughened by high cis-1,4 polybutadiene. Polymers.

[32] Lin Y., Ng K.M., Chan C.M., Sun G., Wu J. (2011). High-impact polystyrene/halloysite nanocomposites prepared by emulsion polymerization using sodium dodecyl sulfate as surfactant. J. Colloid Interface Sci..

[33] Blok L.G., Longana M.L., Yu H., Woods B.K.S. (2018). An investigation into 3D printing of fibre reinforced thermoplastic composites. Addit. Manuf..

[34] García E., Núñez P.J., Chacón J.M., Caminero M.A., Kamarthi S. (2020). Comparative study of geometric properties of unreinforced PLA and PLA-Graphene composite materials applied to additive manufacturing using FFF technology. Polym. Test..

[35] Foster C.W., Down M.P., Zhang Y., Ji X., Rowley-Neale S.J., Smith G.C., Kelly P.J., Banks C.E. (2017). 3D Printed Graphene Based Energy Storage Devices. Sci. Rep..

[36] Caminero M.Á., Chacón J.M., García-Plaza E., Núñez P.J., Reverte J.M., Becar J.P. (2019). Additive manufacturing of PLA-based composites using fused filament fabrication: Effect of graphene nanoplatelet reinforcement on mechanical properties, dimensional accuracy and texture. Polymers.

[37] Gnanasekaran K., Heijmans T., van Bennekom S., Woldhuis H., Wijnia S., de With G., Friedrich H. (2017). 3D printing of CNT- and graphene-based conductive polymer nanocomposites by fused deposition modeling. Appl. Mater. Today.

[38] Al Rashid A., Khan S.A., Al-Ghamdi S.G., Koç M. (2021). Additive Manufacturing of Polymer Nanocomposites: Needs and Challenges in Materials, Processes, and Applications. J. Mater. Res. Technol..

[39] Wei X., Li D., Jiang W., Gu Z., Wang X., Zhang Z., Sun Z. (2015). 3D Printable Graphene Composite. Sci. Rep..

[40] Dul S., Fambri L., Pegoretti A. (2016). Fused deposition modelling with ABS-graphene nanocomposites. Compos. Part A Appl. Sci. Manuf..

[41] Zhu D., Ren Y., Liao G., Jiang S., Liu F., Guo J., Xu G. (2017). Thermal and mechanical properties of polyamide 12/graphene nanoplatelets nanocomposites and parts fabricated by fused deposition modeling. J. Appl. Polym. Sci..

[42] Sieradzka M., Fabia J., Biniaś D., Fryczkowski R., Janicki J. (2020). The Role of Reduced Graphene Oxide in the Suspension Polymerization of Styrene and Its Effect on the Morphology and Thermal Properties of the Polystyrene/rGO Nanocomposites. Polymers.

[43] Ślusarczyk C., Sieradzka M., Fabia J., Fryczkowski R. (2020). Supermolecular Structure of Poly(butylene terephthalate) Fibers Formed with the Addition of Reduced Graphene Oxide. Polymers.

[44] Şahin T., Sinmazçelik T., Şahin Ş. (2007). The effect of natural weathering on the mechanical, morphological and thermal properties of high impact polystyrene (HIPS). Mater. Des..

[45] Kalantari B., Mojtahedi M.R.M., Sharif F., Rahbar R.S. (2015). Effect of Graphene Nanoplatelets Presence on the Morphology, Structure, and Thermal Properties of Polypropylene in Fiber Melt-Spinning Process. Polym. Compos..

[46] Sanes J., Sánchez C., Pamies R., Avilés M.D., Bermúdez M.D. (2020). Extrusion of polymer nanocomposites with graphene and graphene derivative nanofillers: An overview of recent developments. Materials.

[47] Bian J., Lin H.L., He F.X., Wang L., Wei X.W., Chang I.T., Sancaktar E. (2013). Processing and assessment of high-performance poly(butylene terephthalate) nanocomposites reinforced with microwave exfoliated graphite oxide nanosheets. Eur. Polym. J..

[48] Socrates G. (2001). Infrared and Raman Characteristic Group Frequencies: Tables and Charts.

[49] Aumnate C., Pongwisuthiruchte A., Pattananuwat P., Potiyaraj P. (2018). Fabrication of ABS/Graphene oxide composite filament for fused filament fabrication (FFF) 3D Printing. Adv. Mater. Sci. Eng..

[50] Akhina H., Ramya K.A., Gopinathan Nair M.R., Saiter-Fourcin A., Garda M.R., Deshpande A.P., Kalarikkal N., Thomas S. (2020). Influence of reduced graphene oxide on flow behaviour, glass transition temperature and secondary crystallinity of plasticized poly(vinyl chloride). RSC Adv..

[51] Shi J., Yang J., Zhou J., Ji H., Tang X., Gao T. (2020). Effect of graphene on thermal stability and mechanical properties of ethylene-vinyl acetate: A molecular dynamics simulation. Mater. Res. Express.

[52] Rosa F., Négrier P., Corvis Y., Espeau P. (2016). Crystal structure determination and thermal behavior upon melting of p-synephrine. Thermochim. Acta.

[53] Li Z., Reimer C., Wang T., Mohanty A.K., Misra M. (2020). Thermal and mechanical properties of the biocomposites of Miscanthus biocarbon and poly(3-hydroxybutyrate-co-3-hydroxyvalerate) (PHBV). Polymers.

[54] Antich P., Vázquez A., Mondragon I., Bernal C. (2006). Mechanical behavior of high impact polystyrene reinforced with short sisal fibers. Compos. Part A Appl. Sci. Manuf..

[55] Amani M., Sharif M., Kashkooli A., Rahnama N., Fazli A. (2015). Effect of mixing conditions on the selective localization of graphite oxide and the properties of polyethylene/high-impact polystyrene/graphite oxide nanocomposite blends. RSC Adv..

[56] Zare Y. (2016). Study of nanoparticles aggregation/agglomeration in polymer particulate nanocomposites by mechanical properties. Compos. Part A Appl. Sci. Manuf..

