# Supplementary material for: High-Impact Polystyrene Reinforced with Reduced Graphene Oxide as a Filament for Fused Filament Fabrication 3D Printing
Source: Materials (Basel). 2021 Nov 19;14(22):7008. doi: 10.3390/ma14227008 (PMC8623337; doi:10.3390/ma14227008)
Supplement: Supplementary file 1 [file materials-14-07008-s001.zip › materials-1452647-supplementary.pdf]

materials
MDPI

Supplementary information

High-Impact Polystyrene Reinforced with Reduced Graphene Oxide as a Filament for Fused Filament Fabrication 3D Printing

Marta Sieradzka [1,*], Janusz Fabia [1], Dorota Biniaś [1], Tadeusz Graczyk [1] and Ryszard Fryczkowski [1]

[1] Faculty of Materials, Civil and Environmental Engineering, University of Bielsko-Biala, Willowa 2, 43-309 Bielsko-Biala, Poland; jfabia@ath.bielsko.pl (J.F.); dbinias@ath.bielsko.pl (D.B.); tgraczyk@ath.bielsko.pl (T.G.); rfryczkowski@ath.bielsko.pl (R.F.)

* Correspondence: msieradzka@ath.bielsko.pl; Tel.: +48-338279111

Abstract: Graphene and its derivatives, such as graphene oxide (GO) or reduced graphene oxide (rGO), due to their properties, have been enjoying great interest for over two decades, particularly in the context of additive manufacturing (AM) applications in recent years. High-impact polystyrene (HIPS) is a polymer used in 3D printing technology due to its high dimensional stability, low cost, and ease of processing. However, the ongoing development of AM creates the need to produce modern feedstock materials with better properties and functionality. This can be achieved by introducing reduced graphene oxide into the polymer matrix. In this study, printable composite filaments were prepared and characterized in terms of morphology and thermal and mechanical properties. Among the obtained HIPS/rGO composites, the filament containing 0.5 wt% of reduced graphene oxide had the best mechanical properties. Its tensile strength increased from 19.84 to 22.45 MPa, for pure HIPS and HIPS-0.5, respectively. Furthermore, when using the HIPS-0.5 filament in the printing process, no clogging of the nozzle was observed, which may indicate good dispersion of the rGO in the polymer matrix.

Keywords: high-impact polystyrene; reduced graphene oxide; composites; filament for 3D printing

S1. Preparation of samples

S1.1. Graphene oxide preparation

The graphite oxidation proceeded according to the following procedure: 30 g of graphite (< 20 µm) was added into a beaker with 750 cm^3 of sulfuric acid(VI). The mixture was stirred for 1 h at room temperature. After that time, the beaker was placed in an ice bath to lower down the temperature of the reaction mixture (below 10 °C). After cooling, $KMnO_4$ was batched into the beaker. Then, the mixture remained in the ice-bath for 10 minutes. The oxidation reaction continued for 2.5 h, not exceeding 40 °C. After that time, the samples were washed with distilled water (750 cm^3), warm distilled water (60 °C, 600 cm^3) and 3% aqueous solution of H_2O_2. The obtained graphene oxides were purified to remove ions coming from the reagents used. For this purpose, GO was washed several times with distilled water and 10% aqueous solution of hydrochloric acid. The obtained graphene oxide was partially dried.

S1.2. Reduced graphene oxide preparation

The prepared graphene oxides were thermally reduced by micro-explosion. For this purpose, graphene oxide was placed in a thermal reduction chamber that was blown with an inert gas (nitrogen). The graphene oxide in the chamber was heated at a rate of approximately 30 °C/min until micro-explosion occurred. The thermal reduction was carried out until the process ceased to take place in a rapid way. The procedure described above was repeated five times, yielding sample of reduced graphene oxide (rGO).

S2. Characterization of reduced graphene oxide

Before the introduction of rGO into the polymer matrix, the nanoadditive was characterized by the WAXS, FTIR, EDS, and TGA studies.

WAXS pattern of reduced graphene oxide is shown in Fig. S1a. A broad and low intensity peak, characteristic for rGO, appears at an angle of $2\ \Theta$=23.9°. Based on Bragg's equation, $n\lambda=2d\ \sin\theta$, where n is an integer, λ is the wavelength, d is the interlayer spacing, and θ is the scattering angle, the distance between the layers for rGO was determined and is 0.37 nm.

The characteristic bands for rGO are visible in the FTIR spectrum (Fig. S1b). Although the thermal reduction was conducted, the bands for carbonyl (at 1745 cm^{-1}) and carboxyl groups (at 1290 cm-1), which are described as more stable oxygen-functional groups are still present. The band at 1622 cm^{-1} corresponding to the aromatic C=C ring stretching can be found. These results show that low-temperature thermal reduction is an efficient way to remove less stable oxygen-containing functional groups from graphene oxide. The above results were confirmed by EDS analysis (Fig. S1c).

The two-step weight loss is observed on the thermogram for reduced graphene oxide in Fig. 1b. At temperatures up to 400 °C, the sample loses only about 6% of its weight.

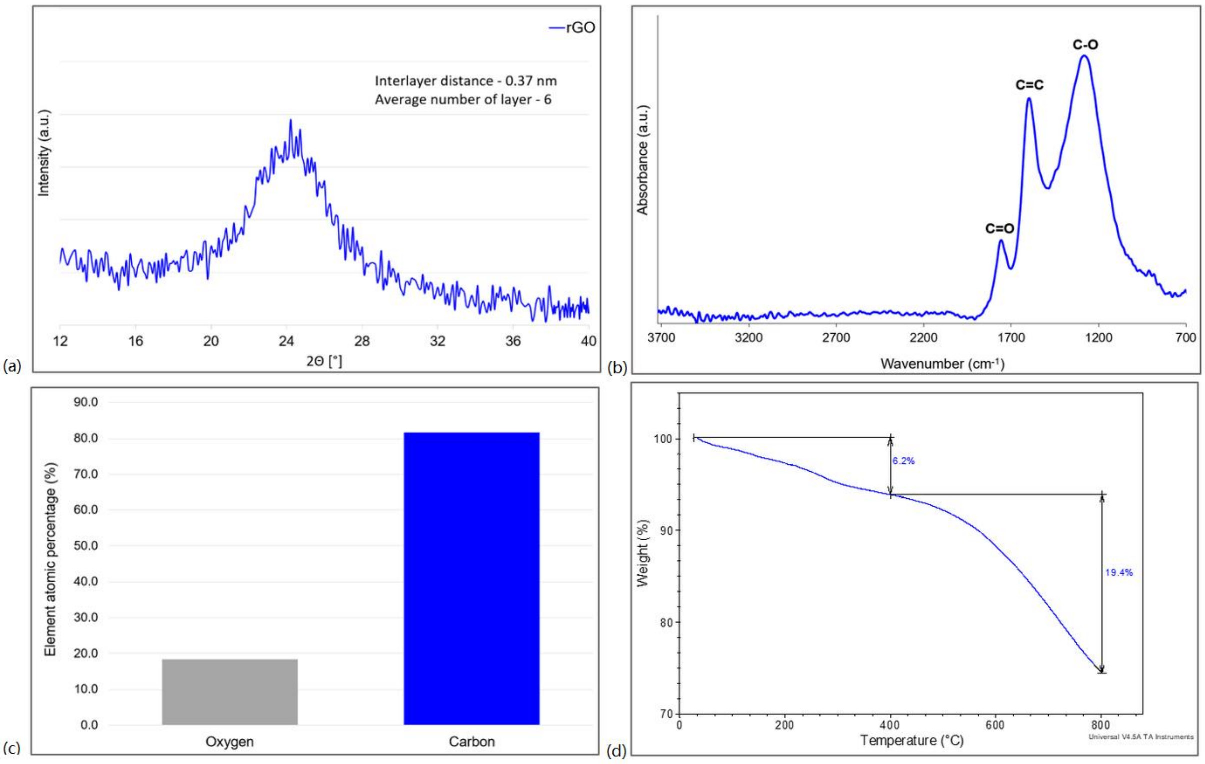

Figure S1. Characterisation of reduced graphene oxide: WAXS pattern (**a**), FTIR spectra (**b**), EDS analysis (**c**), TG curve (**d**).

S3. Mechanical properties of printed parts

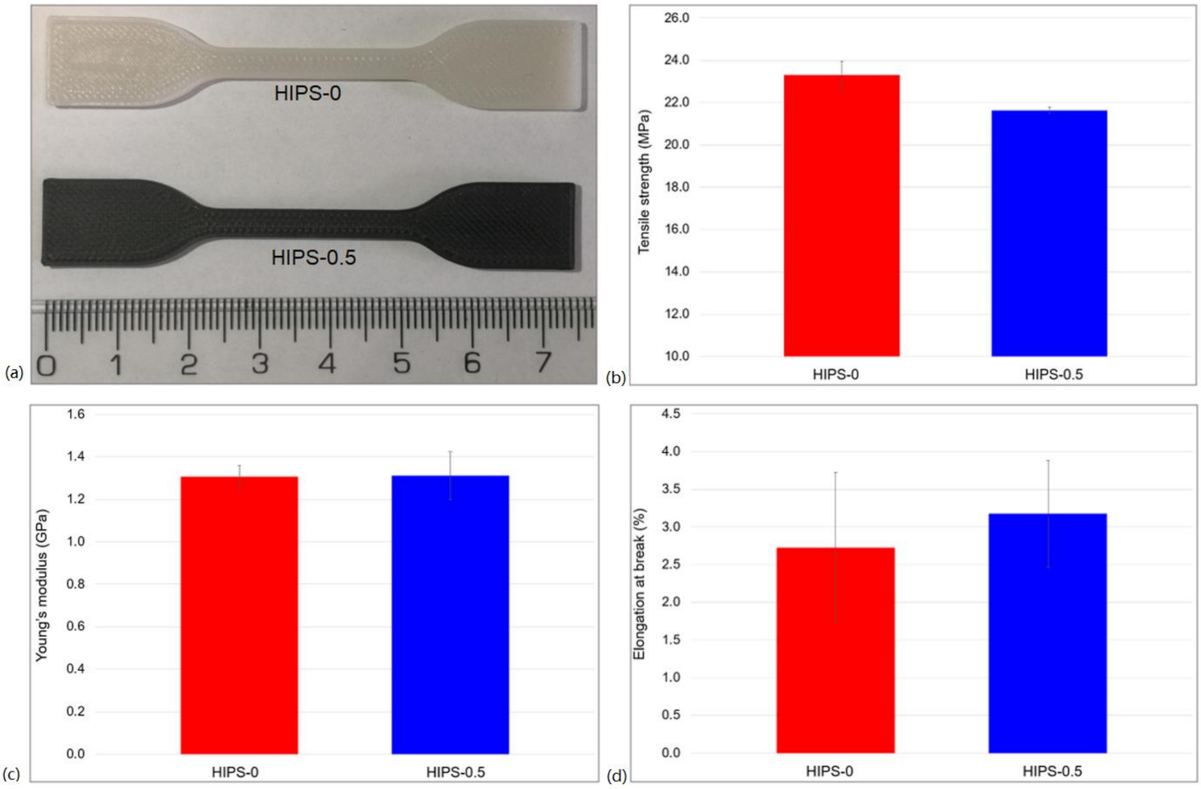

Figure S2. Picture of HIPS-0 and HIPS-0.5 tensile bars (**a**), the value of tensile strength (**b**), Young's modulus (**c**), and elongation at break of printed parts (**d**).
